# Age-related modifications in CYP-dependent drug metabolism: role of stress

**DOI:** 10.3389/fendo.2023.1143835

**Published:** 2023-05-24

**Authors:** Maria Konstandi, Elizabeth O. Johnson

**Affiliations:** ^1^ Department of Pharmacology, Faculty of Medicine, School of Health Sciences, University of Ioannina, Ioannina, Greece; ^2^ Department of Anatomy, School of Medicine, European University of Cyprus, Nicosia, Cyprus

**Keywords:** drug metabolism, cytochrome, CYP3A4, CYP2D6, age, stress ageing and CYP-dependent drug metabolism

## Abstract

Accumulating clinical evidence indicates extensive inter-individual variations in the effectiveness and adverse effects of standard treatment protocols, which are largely attributed to the multifactorial regulation of the hepatic CYP-dependent drug metabolism that is connected with either transcriptional or post-translational modifications. Age and stress belong to the most important factors in CYP gene regulation. Alterations in neuroendocrine responses to stress, which are associated with modified hypothalamo-pituitary-adrenal axis function, usually accompany ageing. In this light, ageing followed by a decline of the functional integrity of organs, including liver, a failure in preserving homeostasis under stress, increased morbidity and susceptibility to stress, among others, holds a determinant role in the CYP-catalyzed drug metabolism and thus, in the outcome and toxicity of pharmacotherapy. Modifications in the drug metabolizing capacity of the liver with age have been reported and in particular, a decline in the activity of the main CYP isoforms in male senescent rats, indicating decreased metabolism and higher levels of the drug-substrates in their blood. These factors along with the restricted experience in the use of the most medicines in childhood and elderly, could explain at an extent the inter-individual variability in drug efficacy and toxicity outcomes, and underscore the necessity of designing the treatment protocols, accordingly.

## Introduction

To date, the outcome of pharmacotherapy remains a complex and challenging issue. Over the years, accumulating clinical evidence indicates that each patient represents an individual case in drug treatment. This notion is mainly based on the extensive inter-individual variations observed in the efficacy of standard protocols used in the treatment of diseases, such as depression, cancer, hypertension, epilepsy and diabetes, as well as in the drug-related adverse reactions and toxicity outcomes (1–4). In particular, when a multi-drug therapeutic scheme is followed, the failure of pharmacotherapy and drug toxicity is more likely to occur. The diversity in the drug response among individuals is largely attributed to the multi-factorial machinery that regulates the biological activity and fate of a drug in the body. Genes that encode factors holding determinant roles in cell signaling, metabolic and transport processes, participate in this machinery. It is well defined that these genes are regulated by various factors including age, gender, race, stress, disease, drugs, diet, lipidemic and endocrinological state, among others (2, 5–8). It should be noted also that important modifications in the functional integrity of the cardiovascular, immune, respiratory, gastrointestinal, central nervous and endocrinological systems take place as we grow older, which have a decisive impact on the factors controlling drug activity and therefore, the outcome and toxicity of pharmacotherapy (1–3, 5, 8–10). This is mainly due to the determinant roles these factors play in the regulation of the absorption, distribution, metabolism, excretion and activation of the drugs. In this regulatory loop, stress among other factors, holds a key role in the regulation of several enzymes catalyzing the metabolism of the majority of prescribed medicines (8, 11) (2, 4, 12–15). The age-related modifications in vital functions of the body and stress perception over the years along with the lack of experience in the use of drugs in children and old people, may explain at least in part, the important deviations in drug efficacy and toxicity observed in childhood and elderly (16); (https://www.msdmanuals.com/professional/geriatrics/drug-therapy-in-older-adults/pharmacokinetics-in-older-adults).

## Drug Metabolism

Every drug entering the body is recognized as a threat of homeostasis and the detoxifying systems are activated (2, 17). The liver is the major site of drug metabolism, where various enzymes catalyze specific metabolic reactions including oxidation, reduction, hydrolysis, hydration, conjugation, condensation, or isomerization aiming at formation of water soluble molecules that can be readily excreted in urine and bile (2, 18).

During drug metabolism of Phase I, cytochrome P450s (CYPs), flavin-containing monoxygenases (FMO) and epoxide hydrolases (EH) are the main families of enzymes that catalyze the biotransformation of xenobiotics including drugs. In Phase II, the metabolic products of Phase I are conjugated with glucuronic acid, glutathione, sulphate and acetyl groups to form highly water soluble complexes, a process that facilitates and accelerates the detoxification of the organism (19). These conjugation reactions are catalyzed by glucuronosyl-transferases (UGT), glutathione S-transferases (GST), UDP- sulfo-transferases (SULT) and N-acetyl-transferases (NAT), respectively (19). Long-term disturbances of these metabolic processes usually result in intracellular accumulation of metabolites and free radicals that could trigger toxic manifestations (18).

The structure of the drug determines whether one or more of these enzymes will be involved in its metabolism during Phase I. Usually, metabolic biotransformation of a drug modifies drastically the drug’s pharmacokinetic, pharmacodynamic and potentially, toxicity profiles (2, 5, 19–22). In most cases, the metabolism of drugs at Phase I leads to their inactivation. Nonetheless, it is also possible that some metabolites are pharmacologically active, and sometimes they are even more active than the parent compound (2, 8). It should be noted that several active metabolic products may have the potential to induce toxic manifestations including cell death, oxidative stress, tumor initiation and teratogenesis, among others (4, 19, 20, 23–26). There are also inactive or weakly active drugs called pro-drugs, which are converted to pharmacologically active metabolites within the body through metabolic reactions predominantly catalyzed by cytochromes (8, 19, 27–30).

The extensive variation among different patients and populations in the hepatic drug metabolic rate is of paramount clinical interest. The spectrum of factors holding major roles in this diversity includes genetic factors, comorbidities, such as chronic liver disorders, diabetes, advanced heart failure and cancer, as well as drug interactions and mainly those resulting in the acceleration or inhibition of drug metabolism (2, 4, 8).

## CYP-catalyzed drug metabolism

CYPs are hemoproteins, which are considered as the most important enzymes in animals and humans that catalyze during Phase I the metabolic biotransformation of the majority of prescribed drugs. They can recognize and subsequently metabolize numerous structures, as they have broad and overlapping substrate specificities. The main CYP isozymes are expressed in all tissues, and predominantly in the liver, where they display the highest capacity. Based on their amino acid sequence homology, the main CYP isozymes that are involved in the metabolism of drugs are arranged into three gene families (CYP1, CYP2 and CYP3) (19, 21, 31). Among the hundreds of CYPs, the CYP1A2, CYP2A6, CYP2B6, CYP2C8/9/19, CYP2D6, CYP2E1 and CYP3A4 are considered as the most important human CYP isoforms (21, 32–34) that catalyze diverse oxidation reactions, such as heteroatom oxidations, hydroxylations, epoxidations, heteroatom dealkylations, oxidative group transfer, cleavage of esters, and dehydrogenations (22, 35). CYPs including CYP2D6, also catalyze the biosynthesis or catabolism of steroid hormones, fat-soluble vitamins, bile acids, fatty acids, eicosanoids and neurotransmitters (23, 36).

Genetic polymorphism and mutations of CYP genes including CYP1A1, CYP2A6, CYP2A13, CYP2C8/9/19, CYP2D6, CYP2B6 and CYP3A4 (19, 37, 38) and the variations in the distribution of the common CYP gene allelic variants that are observed among several ethnic populations, such as Africans, White and African Americans, Asians, Caucasians and Europeans, are among the predominant factors determining to a great extent, the inter-individual and inter-ethnic deviations in drug response and adverse reactions (39, 40). Based on CYP polymorphism, populations could be divided into four phenotypes: the ultra-rapid metabolizers (UM), who display more than two active CYP genes, the extensive metabolizers (EM), who carry two functional CYP genes, the poor-metabolizers (PM), who lack a functional CYP isozyme due to deleted or defective CYP gene and the intermediate metabolizers (IM), who carry one functional and one defective CYP gene or two defective CYP genes. UM cannot reach therapeutic concentrations of drugs in blood and tissues following the usual dosing regime, because they metabolize rapidly the drugs-substrates, whereas PM display slow drug-substrate metabolism that results in the accumulation of the drug in the blood even at usual doses, a condition that may favor toxic manifestations (2). Apparently, CYP gene polymorphism determines a drug’s pharmacokinetic and pharmacodynamic profile (18). From a clinical perspective, the pharmacogenetic profile of a patient in terms of CYP polymorphism could be implemented in improving the outcome of pharmacotherapy and reducing drug toxicity (5, 9, 39).

## Stress impact on CYP-catalyzed drug metabolism

Various external and internal factors can modify the regulation of most CYP *genes*. They can either induce or inhibit the expression of a particular *CYP* resulting in acceleration or inhibition respectively, of the metabolism of its drug-substrates thus modifying accordingly, the drug’s pharmacokinetic and toxicity outcomes. Among these factors, stress holds a determinant role in the expression and activity of the main CYP isozymes that catalyze the metabolism of the majority of drugs in the market. In our preclinical studies several animal models of stress were employed, such as the mild unpredictable stress for 7 days, the repeated restraint stress (2hrs per day x 4 days) and the maternal deprivation stress for 24hrs. The findings indicated that mainly restraint stress and to a lesser extent maternal deprivation stress can modify constitutive and induced expression levels of CYP3A, CYP2C, CYP2D and CYP1A at an extent that could affect a drug’s pharmacokinetic profile (2, 4, 8, 11); https://www.fda.gov/drugs/drug-interactions-labeling/drug-development-and-drug-interactions-table-substrates-inhibitors-and-inducers).

**Figure 1:**
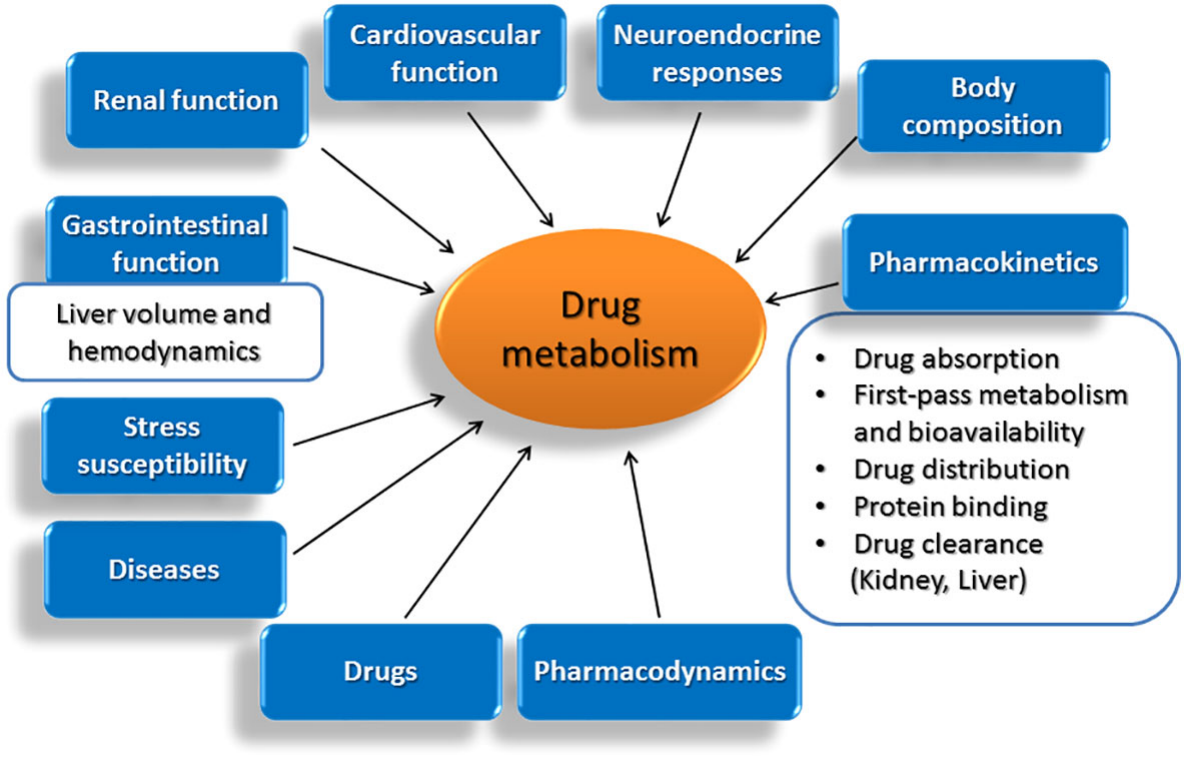
Age-related modifications in vital functions of the body and in other parameters that affect decisively the drug metabolizing capacity of the liver.

It is well documented that the stress-mediated effect on each particular CYP *gene* is stress-specific involving various mechanisms, such as transcriptional regulation by ligand-activated nuclear receptors including PXR (pregnane X receptor), CAR (constitutive androstane receptor) and AhR (aromatic hydrocarbon receptor) (2, 8, 11, 19, 41). In the stress-mediated regulation of CYP *genes*, the stress-system effectors, epinephrine and norepinephrine released from adrenal medulla and glucocorticoids released from adrenal cortex, hold central roles by activating major signal transduction pathways in the liver, such as the AR/cAMP/PKA, JNK, growth hormone (GH)/signal transducer and activator of transcription 5b (STAT5b), PI3K/AKT/FOXO1β and Glucocorticoid/GR-linked pathways. Activation of these pathways usually results in up-regulation of several transcription factors including hepatocyte nuclear factor 4α (HNF4α), hepatocyte nuclear factor 1α (HNF1α), CAR, PXR, RXR, AhR and peroxisome proliferator activated receptor α (PPARα) that hold determinant roles in CYP regulation (**Figure 1**; (2, 13, 14, 42–46). It should be noted that prolonged activation of these pathways can result in accumulation of free radicals and other toxic metabolic products in tissues, which along with the stress-mediated reduction of glutathione content, constitute a condition that usually favors the development of severe disease states (2, 4, 19, 47). Within the spectrum of the stress-induced events, alterations in the secretion of hormones, such as GH, prolactin (PRL), thyroid hormones and insulin, the increased release of cytokines/NF-kB and oxidative stress, play determinant roles in CYP regulation by stress (2, 7, 8, 48, 49), (**Figure 1**). In particular, activation of the hypothalamo-pituitary-adrenal (HPA) axis by stress results in the somatostatin-mediated inhibition of thyroid-releasing hormone (TRH) and thyroid-stimulating hormone (TSH) release and in reduced conversion of thyroid T4 to T3 (active hormone) that in humans, displays a negative control on various CYP genes including those belonging to the CYP3A subfamily (50). It is well defined that the stress-induced somatostatin release and adrenergic receptor stimulation inhibit GH secretion (51–54), an effect of paramount significance in drug metabolism, because GH, among other effects, positively regulates various CYPs, such as CYP3A4 (55), CYP2C and CYP2D (7, 48, 56, 57) and its up-regulating effect is mediated by the GH-pulse activated transcription factor, STAT5b (7, 23, 56, 57). Notably, GH suppression is followed by down-regulation of the insulin-like growth factor (IGF-1), which holds a determinant role in the insulin-mediated down-regulation of various CYPs including CYP3A4, CYP2C, CYP1A and CYP2D (2, 46, 49, 58–60). It is known that stress reduces PRL release (61), which down-regulates several CYPs including those of CYP1 family (62, 63). The effects of glucocorticoids on drug-metabolizing enzymes derive from a combination of distinct mechanisms involving their stimulating effects on the synthesis and activity of several transcription factors, such as CAR, PXR, RXR, AhR, HNF4α and STAT5b, critical CYP regulators (64, 65). It is of interest also to note that the stress-induced release of adrenaline stimulates alpha- and beta-adrenergic receptor-linked pathways and in turn, the release of cytokines, IL-1β, IL-6 and TNFα, that down-regulate hepatic CYP3A and CYP2C (66–68).

The impact of stress on drug metabolism is unique and distinct from that of medicines that usually display dose- and time-dependent specificities (2, 8, 11). Specifically, repeated restraint stress repressed hepatic *CYP2E1* and *CYP2B* constitutive expression, whereas it up-regulated most of the other CYPs including those belonging to CYP1A, CYP2A, CYP2C, CYP2D and CYP3A subfamilies and metabolize over 70% of prescribed drugs (2, 8, 22, 69). It is of note that CYP1A1 and CYP1A2 substrates including several drugs and pre-carcinogens, such as polycyclic aromatic hydrocarbons (PAHs), act as ligands of AhR and induce these CYP1A isozymes (31) thus accelerating their metabolism. On the other hand, inhibition of these isozymes can result in reduced metabolism of their drug-substrates and restricted bioactivation of inactive pre-carcinogens (70–72). In terms of the impact of stress on CYP2D expression, it appears that repeated restraint stress induced CYP2D that to date was considered as a non-inducible enzyme. CYP2D catalyzes the metabolism of most antidepressants, anxiolytic, antipsychotic and antiepileptic drugs along with several antiarrythmic drugs, beta- and calcium channel-blockers (69, 73, 74). The role of CYP2D in the synthesis of dopamine, serotonin and neurosteroids in the brain is well defined and the reason of the current intensive investigation on its role in Parkinson’s disease and other neurodegenerative disorders (4, 75–78). From a clinical perspective, the stress-induced up-regulating effect on the afore-mentioned CYPs could result in acceleration of the metabolism of their drug-substrates and therefore, in sub-therapeutic drug levels in blood and tissues (2, 4, 8). On the other hand though, the stress-induced CYP up-regulation could increase the activation of pro-drug-substrates and therefore, the drug efficacy, whereas the opposite is true for the stress-mediated CYP down-regulation (2, 4, 8).

There is also an indirect involvement of stress in drug metabolism as long-term exposure to uncontrolled stress may trigger several pathophysiological states including cancer, depression, inflammatory diseases, diabetes mellitus and other diseases within the spectrum of metabolic syndrome, which comprise modified hormonal, nutritional, immune and psychological states compared to healthy population (43, 44), condition that has been largely associated with the patient’s hepatic drug metabolizing capacity (2, 4, 8). Based on the accumulating evidence that stress and the major effectors of the stress response, epinephrine and glucocorticoids, play significant and distinct roles in the regulation of the metabolism of numerous drugs in the market, clinicians should consider the stress profile of the patient when designing treatment protocols and dosing regimes. Although stress can not be included in any treatment algorithm, as it does not display the dose- and time- dependent properties of a drug, it is suggested that stress should be alleviated in patients, in order to assure the highest drug efficacy with the less possible adverse reactions (2, 4, 8). Furthermore, drugs with sympathomimetic properties or adrenergic receptor blockers, or those who modify GH, thyroid hormone, insulin and glucocorticoid hormone status should be considered in treatment protocols, because they can decisively modify the pharmacokinetic and pharmacodynamic profiles of drugs and therefore, the efficacy and toxicity of pharmacotherapy (2, 4, 8).

## Age-related modifications of critical parameters determining hepatic drug metabolism

Although a comprehensive definition of ageing is not possible, several characteristic alterations including the time-related loss of functional units of organs, the disruption of functional integration between cells and organs and the failure in preserving homeostasis under stress are recognized. In this light, ageing is followed by a progressive accumulation of random modifications in the function of vital systems in the body, which take place at molecular, cellular and tissue level and are followed by decreased viability and increased morbidity (79). Within the spectrum of modifications observed with age, a decline in the functional integrity of organs, such as heart, kidneys and liver, holds central role. Among others, ageing is followed by a progressive reduction in liver volume and blood flow, parameters that drastically affect hepatic drug metabolism. Within pharmacokinetic implications occurring with age and affect the fate and activity of drugs in the body, the composition of the body is included (80), along with alterations in drug absorption, first-pass metabolism and bioavailability (81–84), drug distribution (80, 85), protein binding (79, 86) and drug clearance (79, 87–91). Furthermore, age-related diseases including congestive heart failure and hypertension and the drugs used for their treatment, can also modify drug pharmacokinetics (79, 85, 92–95).

It should be noted that alterations in neuroendocrine responses to stress, which are associated with modified HPA axis function, usually accompany ageing (79). In the aged-brain and in particular, the hippocampus, increased oxidative stress and reduced synthesis of neurotrophins are observed (96, 97). These ageing-dependent alterations in the limbic system can affect critical biobehavioral parameters, such as emotions and coping with stress (98). Notably, susceptibility to stress increases with age *via* up-regulation of the NADPH oxidase in the hippocampus (99). It should be also taken into account the fact that aged people exhibit increased plasma glucocorticoid levels (GC), which along with epinephrine and norepinephrine organize the response of the stress system to stress stimuli (42, 43, 45, 53, 99). Interestingly, the dysfunction of the stress-regulating neuroendocrine system in elderly people along with the ageing-dependent oxidative stress are equivalent to those in humans exposed to chronic stress (99, 100). Apparently, elderly people are more vulnerable to chronic stress that determines among other parameters, the age-related dynamics in the hepatic CYP-dependent drug metabolism and therefore, the drug efficacy and toxicity (2, 99).

## Age-related modifications in the CYP-catalyzed drug metabolism

It is worth noting that about 50% of drugs are prescribed by clinicians without following age-related guidelines and in only 10% of drugs administered in neonates and infants the safety and efficacy has been evaluated. The age-related variations in the activity and toxicity of drugs depend largely on the metabolic capacity of the liver of patients, which is clinically very important, because it determines a drug’s pharmacokinetic and pharmacodynamic profile. Immaturity of drug-metabolizing systems in children and their decline in elderly, have been associated with increased risk of drug toxicity (101).

The drug metabolizing systems are under continuous modifications during our lifespan and this fact should be considered when prescribing drugs in children and the elderly. In particular, ageing is followed by significant modifications in the hepatic CYP isozyme expression pattern in humans, which are CYP isozyme-specific (102) and these modifications can affect drastically the oxidative biotransformation of the majority of drugs in the market, and numerous other xenobiotics including environmental pollutants, pre-carcinogens, carcinogens, and toxicants along with the biosynthesis of several endogenous compounds (101, 102).

There are several studies reporting considerable variations in the hepatic ontogenic gene expression pattern of the major CYPs in children and the elderly. These variations may underlie the fact that children are often less responsive to drugs and are exposed to higher risk in developing adverse reactions related to drugs (103). Of particular interest is the fact that maturational changes in CYP3A ontogeny may affect the clinical outcome of many drugs (~50% of drugs in the market) (104). Therefore, the impact of ontogeny on CYP3A and other CYP isozymes should be considered by the clinicians when prescribing drugs-substrates, inducers or inhibitors of these CYPs (104). But, variations in CYP-catalyzed drug metabolism among individuals at different ages is not only related to ontogeny, but also to exposure to environmental pollutants, the diet, the gender and genetic polymorphism often observed within different ethnicities (2, 6, 103).

Several studies reported an age-related decline in the clearance of medicines undergoing CYP-catalyzed biotransformation in the liver (102, 105–118). In particular, preclinical studies indicated that the CYP-dependent drug metabolism is reduced by about 37-60% in the liver of senescent rats (102, 115, 116, 118, 119). It is also of interest to note that constitutive *CYP1A1* expression was detected only in the livers of 3-week-old rats, whereas it was not detectable in older rats (120–122). In humans also, *CYP1A1* is constitutively expressed only at early stages of development, while it is not detectable in adults (121).

*CYP1A2*, *CYP2B1* and *CYP2E1* were detected at their highest basal protein expression levels in 3-week-old rats, while they were decreased in older rats aged 12- and 26-weeks (122). Hepatic *CYP2B1* was further repressed in old rats aged 104 weeks. Similar age-related variations were observed in the CYP1A1-dependent EROD, the CYP1A2-dependent MROD, the CYP2B1/2-dependent PROD and the CYP2E1-dependent PNP activity levels (102, 122, 123).

Hepatic *CYP2C11* and *CYP3A2* constitutive protein expression and the CYP3A1/2-dependent midazolam activity were drastically induced after puberty in the adulthood and started declining with ageing (122). It was also reported that CYP2C11, the main gender-specific steroid 2α- and 16α-hydroxylase, was not detectable in newborn rat livers. Nonetheless, it was induced at puberty only in males and not in females (124–126). In contrast, the CYP3A1/2-dependent steroid 6β-hydroxylase activity was detectable in the liver of pre-pubertal male and female rats and *CYP3A2* protein expression increased until the age of 12-weeks, when it started declining. Interestingly, *CYP3A2* expression levels were lower in female rat livers at puberty compared to those at a pre-pubertal state. Apparently, there is an age- and gender-dependent regulation of both, *CYP2C11* and *CYP3A1/2* expression patterns in the liver of rats (122, 124–126). Notably, the developmental expression pattern of CYP2C11 in males and females is determined by the gender differentiated GH secretion pattern, but this is not the case for CYP3A2 (57, 102, 125).

There are controversial reports on the effect of ageing on the CYP-dependent drug metabolism in humans. In several studies, no significant modifications of specific CYP isozyme expression patterns were observed in the liver of subjects aged between 12 and 73 years (102, 127, 128). In contrast, there are studies reporting that hepatic CYP2E1 and CYP3A content decreased with age (102, 129). Based on data coming from studies employing specific CYP isozyme substrates, it appeared that the rate of CYP1A2 and CYP2C19 drug-substrate metabolism decreases with age, whereas the CYP3A4-, CYP2A6-, CYP2C9- and CYP2D6-dependent substrate metabolism is slightly or not modified (102, 130, 131). As previously mentioned, hepatic CYP1A1 and CYP1A2 expression patterns are similarly modified with age in rats and humans (122).

Fluctuations in hepatic CYP expression and activity patterns observed during development and with age occur at post-transcriptional level and could be profoundly associated with alterations in GH, gonadal hormones, PRL, thyroid hormone, insulin, glucagon and glucocorticoid levels, because these hormones hold determinant roles in CYP regulation. In particular, GH and gonadal hormones determine sex-differentiation in the *CYP* expression patterns (2, 8, 11, 37, 48, 57, 102, 125, 127, 132–136). In this light, the modified *CYP* expression patterns observed in pregnancy, at different phases of the estrous cycle (137, 138), in menopause or in subjects following hormonal replacement therapy (137, 138) and in diabetes could be explained (102). The age-related alterations in the hepatic CYP expression pattern may also take place at transcriptional level and are often associated with oxidative stress and HNF1α levels, factors that are induced during the metabolic biotransformation of drugs at Phase I (102, 118, 139, 140).

In drug metabolism, CYP inducibility is a significant parameter. Numerous structurally diverse foreign substances and endogenous compounds can induce several CYP genes, resulting in increased synthesis and activity of the corresponding enzymes and acceleration of the drug-substrates metabolism. Most of CYP genes belonging to families 1-4 can be transcriptionally induced by xenobiotics. In this process, cytosolic AhR and the nuclear receptors CAR, PXR and PPARα (peroxisome proliferator-activated receptor α) hold central roles (2, 11, 60, 102, 141–143).

Regarding the effect of age on CYP induction in humans and rats there are controversial reports (102, 144, 145). Several studies reported decreased (102) or no modified CYP induction by various compounds with age in rats (146). In particular, the phenobarbital-induced effect on several CYP genes including CYP3A and CYP2B, was either decreased with age (147) or remained unaffected (148). It is of note though that the CYP3A inducibility by dexamethasone was strongly reduced with ageing (149–151).

## Discussion

Long-term clinical experience demonstrates the high frequency of failure in the treatment protocols followed in pediatric and geriatric patients. This is due in part, to the fact that clinical trials usually engage adults 18-65 years old (152) and thus, the results drown from these studies often do not reflect the potential activity and side effects of a drug in children and elderly patients. This review has focused mainly on alterations in drug metabolism in the elderly, because there is limited information about alterations in drug metabolism at early stages of life including childhood. In addition, there is markedly higher incidence of morbidity with age (over 80% of old people suffer at least from one chronic disease). In comparison to younger individuals, the elderly take about three times more medications and usually, they follow multi-drug treatments (polypharmacy) (153–155), a condition that constitutes a major challenge for health professionals and patients, who often experience memory impairment and either forget to take their medication or take it multiple times (156). It should be noted also that ageing is accompanied by significant alterations in the functional capacity of several organs, such as the liver, kidneys, heart, lungs and intestine, among others (157). All these parameters hold determinant roles in the regulation of drug metabolism in the elderly and may explain the higher incidence of drug-drug interactions and increased incidence of adverse effects and toxicity in old patients (158, 159). It is estimated that about 10% of hospital admissions in the elderly are associated with drug side effects (10, 102, 160–162).

The effects of a drug in the body depend on various factors including body composition, age, stress, gender, race, diet, medication, hormonal state, pathophysiological states, long-term alcohol consumption and smoking that modify fundamental processes determining a drug’s pharmacokinetic and pharmacodynamic profiles (2, 4, 137). In particular, hepatic drug metabolism, mainly at Phase I, is a determinant parameter for drug effectiveness and toxicity and hence, the inter-individual variability in drug metabolism is now considered as a crucial factor in the age-dependent variations in pharmacokinetics and idiosyncratic drug toxicities observed in patients (163, 164); (www.fda.gov/cber/gdens/popharm.pdf).

As previously mentioned, several hormones including GH, cortisol, insulin, PRL and thyroid hormones play important roles in CYP regulation (2, 7, 48, 51, 148) and therefore, the decline in the functional integrity and activity of the hormonal systems that follows ageing is of paramount significance for the pharmacotherapy in elderly patients. Among these age-related modifications, the impairment in HPA axis feedback inhibition with age, which is followed by increased plasma cortisol levels, holds a prevalent role and may explain the increased stress susceptibility in geriatric patients (165). All these modifications observed with age can drastically affect several drug metabolizing enzymes and mainly, several CYP isozymes that catalyze the metabolism of numerous prescribed drugs (2, 4, 11, 51).

In conclusion, the restricted experience in the use of most drugs in children and elderly patients, the decline in memory with age along with the increased stress susceptibility and the decreased activity of various hormonal systems, which follow ageing and largely determine the CYP-dependent drug metabolism, are exceptionally important parameters that should be taken into account when designing treatment regimes in pediatric and geriatric patients, in order to provide them with the appropriate drug dosing regimes and assure the optimal effectiveness and restricted adverse reactions of pharmacotherapy.

## Author contributions

MK drafted the original manuscript and EJ critically revised it. Both authors agree for the content of the work and approved it for publication. All authors contributed to the article and approved the submitted version.
